# Publication Trajectories of Today's Canadian Academic Plastic Surgeons: A Bibliometric Analysis

**DOI:** 10.1177/22925503251371051

**Published:** 2025-08-29

**Authors:** Norbert Banyi, Theresa Buchel, Young Ji Tuen, Sophia Shayan, Rebecca Courtemanche, Jugpal S. Arneja

**Affiliations:** 1 Faculty of Medicine, 8166 The University of British Columbia, Vancouver, British Columbia, Canada; 2 Faculty of Science, 37210 The University of British Columbia, Vancouver, British Columbia, Canada; 3 Division of Plastic Surgery, 37210 BC Children's Hospital, Vancouver, British Columbia, Canada; 4 Sauder School of Business, University of British Columbia, Vancouver, British Columbia, Canada

**Keywords:** research productivity, publication trends, surgical education, career trajectory, Productivité de la recherche, Tendances en matière de publications, Formation des chirurgiens, Évolution de carrière.

## Abstract

**Introduction:** The landscape of academic research has evolved notably in recent decades, shifting towards earlier career publications and more interdisciplinary collaborations. This study aims to identify research productivity trends among Canadian academic plastic surgeons. **Methods:** The Web of Science and MEDLINE databases were searched by plastic surgeon names and for each result, the author list position, year of publication, journal, and citation counts were collected. Surgeons’ demographics, including gender and medical school graduation year, were obtained from provincial college websites. Publication rates over a plastic surgeon's career trajectory were analyzed by surgeons’ current decade of practice. **Results:** There were 3661 included entries in our database, corresponding to 2831 unique publications by 245 surgeons (71%, 175/245 men). The median year of medical school graduation was 2002 (SD 12 years). Surgeons in more recent decades of practice (decade 1 or decade 2) published earlier and more frequently per career decade. A wide distribution of publication rates (range 0-66) was found for surgeons currently in their fourth decade of practice. From 2005 to 2020, the number of publications per year increased dramatically, from 36 publications in 2005 to 198 publications in 2020. Citations normalized by years from publication remained stable. The proportion of first authorship decreased from 0.63 and 0.42 in the pre-medicine and educational decades, to 0.09 and 0.08 by the third and fourth decades of practice (p < .001). **Conclusion:** An emerging trend of earlier and increased publications among newer generations of surgeons was seen. Incentives to participate and mentor in research for surgeons gaining seniority are suggested.

## Résumé

**Introduction :** Le paysage de la recherche universitaire a considérablement évolué au cours des dernières décennies, basculant vers des publications plus tôt en cours de carrière et davantage de collaborations interdisciplinaires. Cette étude vise à identifier les tendances de productivité de la recherche parmi les chirurgies plasticiens universitaires au Canada. **Méthodes :** Une recherche a été menée dans les bases de données Web of Science et Medline à partir des noms de chirurgiens plasticiens et, pour chaque résultat, leur place dans la liste des auteurs, l’année de publication, le nom du journal et les nombres de citations ont été collectés. Les données démographiques des chirurgiens incluant leur sexe et leur année d’obtention du diplôme d’une école de médecine ont été obtenues à partir des sites Web des collèges provinciaux. Les taux de publications au cours de la carrière du chirurgien plasticien ont été analysés en fonction du nombre actuel de décennies de pratique des chirurgiens. **Résultats :** Notre base de données a inclus 3661 entrées correspondant à 2861 publications uniques de 245 chirurgiens (175 hommes/245, soit 71%). L’année médiane d’obtention du diplôme en école de médecine était 2002 (écart-type 12 ans). Les chirurgiens ayant moins d’années d’exercice à ce jour (1 ou 2 décennies) ont publié plus tôt et plus souvent par décennie de carrière. Un large éventail de taux de publications (écart : 0 à 66) a été observé parmi les chirurgiens qui sont actuellement dans leur quatrième décennie de pratique. Le nombre annuel de publications a augmenté considérablement entre 2005 et 2020, passant de 36 en 2005 à 198 en 2020. Le nombre de citations, normalisé par année de publications est resté stable. La proportion de « Premier auteur » a diminué de 0,63 et 0,42 dans les décennies pré-médecine et éducation à 0,09 et 0,08 au cours des 3^e^ et 4^e^ décennies d’exercice (*P* < 0001). **Conclusion :** Nous avons observé une nouvelle tendance de publications plus précoces en plus grand nombre parmi les nouvelles générations de chirurgiens. Des incitations à participer et des mentors en recherche sont suggérés pour les chirurgiens gagnant en ancienneté.

## Introduction

The current era of academic plastic surgery is marked by increasing pressures to publish.^1^ Recent research has shown that peak productivity occurs progressively earlier in academic plastic surgeons’ careers.^2^ Since the abolition of numeric grades in Canadian medical schools, research involvement has emerged as a metric valued by plastic surgery program directors.^3^ Canadian plastic surgery residency programs now have formal research requirements and publication rates are linked to markers of academic success, including full professorship, endowed positions, and editorial board memberships.^4,5^ Moreover, with increased competition in academic plastic surgery, there is additional pressure for medical students and residents to do research to match into residencies and fellowships.^6,7^ Academic productivity influences career advancement at academic institutions, yet few institutions formally incentivize or require plastic surgeons to publish original research.^8,9^

Despite the shift towards earlier productivity peaks, the ongoing publication efforts of experienced plastic surgeons remain essential. Mentorship plays a crucial role in this context, as experienced mentors guide trainees through the nuances of academic publishing, including strategic authorship positioning and maximizing publication output over time.^5,10^ This guidance is critical because a greater number of publications entering residency and stronger perceived mentorship during residency are associated with a future in academia.^5^ Therefore, it is vital that the current generation of academic plastic surgeons continues to mentor trainees through the research process for the sustainability of the advancement of the specialty.

A study by Spake et al (2021) demonstrated that, in the United States (US), there is a trend toward increasing productivity at much earlier stages in careers, likely due to the rising demands for matching into residency programs.^2^ Unlike the US, Canada does not have grade-based metrics like the US Medical Licensing Exam Step 2, which may exaggerate this trend in the Canadian setting. Other factors identified as correlating with higher academic productivity among US plastic surgeons include obtaining fellowships and serving as a fellowship director.^11^ These trends have also been observed in Canada, where the need for fellowship training to become an academic plastic surgeon has increased.^6^ Additionally, Ngaage et al (2023) described a trend of increased collaboration on plastic surgery articles, which may also lead to increased productivity per surgeon.^12^

The objective of this study is to identify trends in research productivity among Canadian academic plastic surgeons. Secondary objectives include identifying trends between surgeons based on their current decade of practice, exploring the overall numbers of publications over time, authorship positions over a career, collaboration between surgeons, and the journals in which surgeons publish.

## Methods

This study was a retrospective bibliometric analysis of the literature produced by Canadian academic plastic surgeons, prior to October, 2023. Surgeons were grouped by their respective decades of practice to compare and contrast trends between decades. Published original research articles, reviews, letters to the editor, and opinion pieces were retrieved, and a database of publications for all Canadian academic plastic surgeons was created with a methodology analogous to that of Wang et al 2021.^13^ Publication rates and authorship positions over a plastic surgeon's education were analyzed. Publication rates over surgeons’ career trajectories were analyzed by the surgeons’ current decade of practice to describe generational trends and authorship positions. The number of publications and citations per paper over time, irrespective of author, were also analyzed.

### Developing a List of Plastic Surgeons

Surgeons who had a Canadian university appointment were included in our study. A list of surgeons from each of the Canadian Academic Plastic Surgery Program websites was created. The plastic surgery divisions at each of these Canadian academic institutions were contacted in May 2023 to verify and, if necessary, update the list. The universities and corresponding number of surgeons used for the database are as follows: University of Toronto (53), University of Calgary (36), University of British Columbia (29), University of Alberta (23), University of Ottawa (23), Dalhousie University (15), Université Laval (15), University of Manitoba (14), McGill University (13), McMaster University (12), and Western University (12).

### Author Details and Publication Timeline

The gender and year of medical school graduation for each plastic surgeon were extracted from the respective province's college of physicians and surgeons database. The medical school start year was assumed to be 4 years prior to the year of medical school graduation. The publication timeframes were categorized as follows: “Pre-medicine” included studies published before the start year of medical school. The initial decade after starting medical school was designated as the “medical education decade,” while the subsequent periods were referred to as “decade 1,” “decade 2,” “decade 3,” and “decade 4” of professional practice.

### Collating Publications and Database Entries

Publications from each surgeon were identified by searching the Web of Science (WoS) and MEDLINE (PubMed) databases by author term (surgeon name). All searches were done in the period July to October 2023. Results from each search were exported and merged before the removal of duplicates. Two authors independently reviewed the remaining articles for inclusion. Original research articles, reviews, letters to the editor, and opinion pieces were included. All other article types were excluded. A combination of author affiliations (current and previous), middle names, and fields of research was used to evaluate and exclude studies by different authors with the same first and last names.

For each included publication, the following variables were collected: the surgeon's name, the complete author list, the surgeon's position within the author list (first, middle, or last), the publication title, year of publication, journal of publication, and the number of citations. Citation numbers for studies were taken preferentially from WoS and then from MEDLINE. Of note, if the surgeon did not occupy the position of the first or last author, they were categorized as a middle author. PubMed Journal abbreviations were converted back to full names based on the PubMed “Linkout Journal List”. Results from each surgeon were then merged to create the database. As a result, multiple database entries for the same publication exist where multiple surgeons collaborate.

### Data Analysis

Descriptive statistics and visualization were conducted to summarize the data. Kruskal-Wallis testing was used to compare publication rates between the surgeons of different decades of current practice for each time period. Statistically significant differences led to post-hoc pairwise Dunn's tests with Bonferroni-corrected p-values. Chi-square tests analyzed the frequency of authorship positions over time, followed by post-hoc comparisons to assess period differences. End-of-decade projections were estimated by multiplying the average current number of publications by the reciprocal of the average proportion of the decade that has passed.

## Results

### Author Details and Decades of Practice

Our database comprised 3661 entries for 245 surgeons, corresponding to 2831 unique publications. Of 2831 unique studies, 618 (22%) had at least two different surgeons in the database as co-authors on these articles. The median year of medical school graduation was 2002 (SD 12 years), corresponding to a median decade of practice of 2. Seventy-one percent (175/245) of the surgeons were men. Plastic surgeons had an average of 14.9 (SD 19.3) publications at the time of our searches. There were 8 entries for pre-medicine years, 450 entries for the educational decade, 1307 entries for decade 1, 916 entries for decade 2, 637 entries for decade 3, and 328 entries for decade 4. There were an additional 15 entries for the fifth decade by 5 surgeons who are currently in their fifth decade of practice. At the time of data collection, there were 74 surgeons in their first decade of practice, 72 surgeons in their second decade of practice, 44 surgeons in their third decade of practice, 42 surgeons who had been practicing for four decades, and 13 who practiced for 5 decades.

### Research Participation

Of the authors analyzed, 2.86% (7/245; 95% CI: 1.39%–5.78%) had published prior to medical school, while 43.67% (107/245; 95% CI: 37.61%–49.93%) published during their educational decade. In the first decade post-education, 61.22% (150/245; 95% CI: 54.99%–67.11%) of authors had published. The proportion of authors who published in the second decade was 53.80% (92/171; 95% CI: 46.33%–61.11%), while the third and fourth decades showed similar proportions of 63.64% (63/99; 95% CI: 53.82%–72.44%) and 63.64% (35/55; 95% CI: 50.40%–75.34%), respectively. In the fifth decade, the proportion of authors who published declined to 38.46% (5/13; 95% CI: 17.72%–64.21%).

### Publication Trajectories

Surgeons averaged 0.03 pre-medicine publications (95% CI 0-0.45), 1.8 in their educational decade (95% CI 0-9.1), 5.3 in their first decade of practice (95% CI 0-22.6), 5.4 in their second (95% CI 0-27.9), 6.4 in their third (95% CI 0-25.3), and 6.0 in their fourth (95% CI 0-31.0).

**Figure 1.**
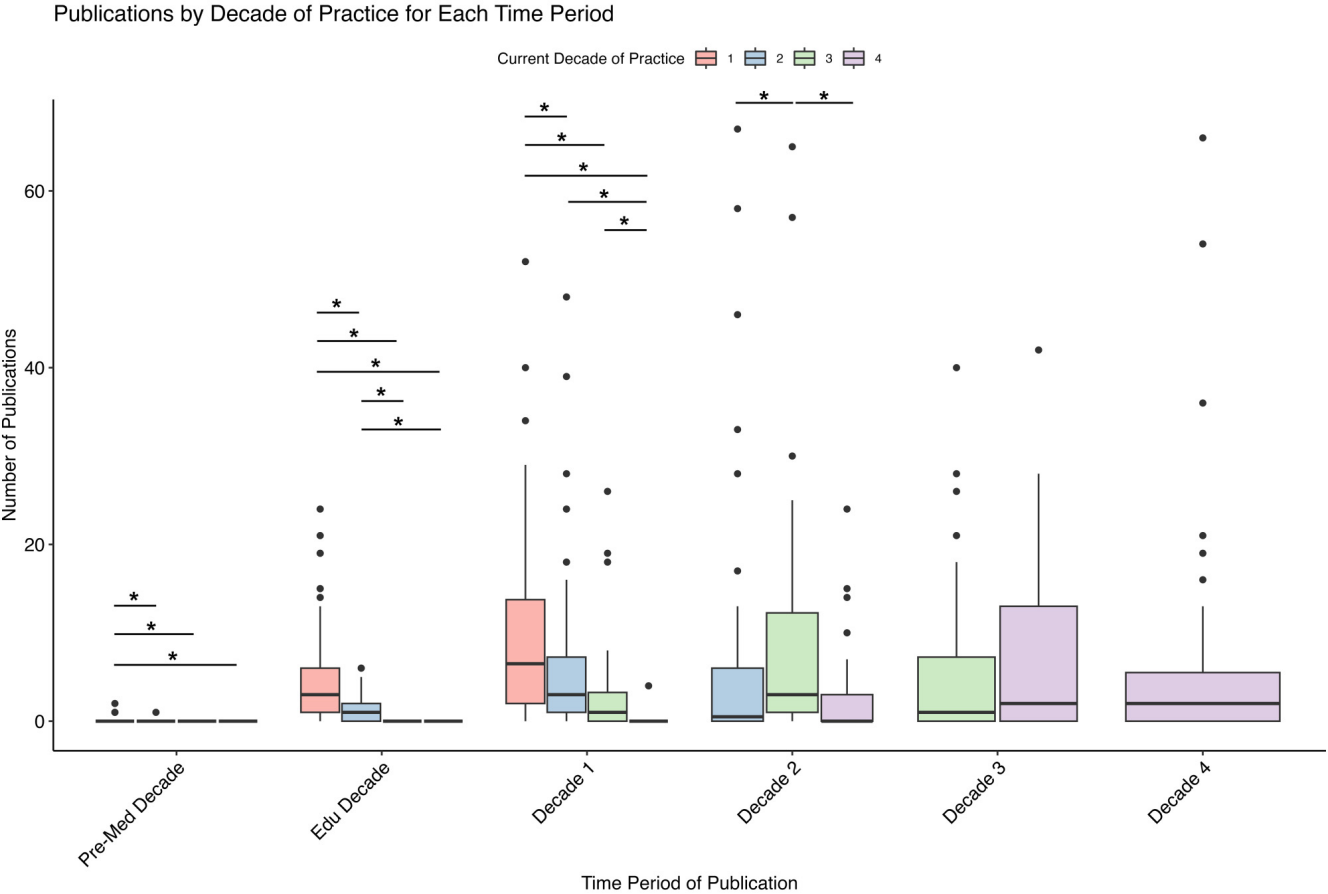
Number of publications per decade where surgeons are stratified by the decade of current practice. There are 74 surgeons 5.7 years on average into their first decade of practice, 72 that are 3.7 years on average into their second, 44 that are 4.2 years on average into their third, and 55 who are 6.6 years on average into their fourth decade of practice.

Figure 1 and Table 1 demonstrate the median number of publications per career decade with surgeons stratified by their current year of practice. There were significant differences between surgeons in different current decades of practice in the following periods: Pre-med (H 10.8, p = 0.01), Educational Decade (H 147.1, p < .001), Decade 1 (H 100.6, p < .001), and Decade 2 (H 15.6, p < .001) (Suppl. Table 1). On post-hoc pairwise testing, in the pre-medicine decade, significant differences were found only between surgeons in their first decade of practice (median 0, IQR 0) and the other groups of surgeons (median 0, IQR 0) due to outliers in the data (Suppl. Table 1). For the educational decade, surgeons in their first decade of practice (median 3, IQR 5) published significantly more than those currently in decade 2 (median 1, IQR 2), decade 3 (median 0, IQR 0), or decade ≥ 4 (median 0, IQR 0) of practice. Likewise, those currently in decade 2 of practice published more than those senior to them in the educational decade (p < .0001). For publications in the first decade of practice, surgeons currently in decade 1 (median 6.5, IQR 11.75) published more than those in decade 2 (median 3, IQR 6.25, p = .036), decade 3 (median 1, IQR 3.25; p < .0001), and decade 4 (median 0, IQR 0; p < .0001). Similarly, in the first decade of practice, surgeons currently in decade 2 published significantly more than those in decade 4 (p < .0001) and surgeons currently in decade 3 published more than those in decade 4 (p = .0001).

**Table 1.** Summary of Publications at Education/Career Stage by Current Decade of Practice.

Parameter	Current Decade of Practice			
	First decade of practice	Second decade of practice	Third decade of practice	≥Fourth decade of practice
Number of surgeons, n	74	72	44	55
Publications by education/ career stage, median [IQR]				
Pre-med	0 [0]	0 [0]	0 [0]	0 [0]
medical education	3 [5]	1 [2]	0 [0]	0 [0]
first decade of practice	6.5 [11.75]	3 [6.25]	1 [3.25]	0 [0]
second decade of practice		0.5 [6]	3 [11.25]	0 [0]
third decade of practice			1 [7.25]	2 [13]
fourth decade of practice				2 [5.5]
Total publications, median [IQR]	10.5 [16]	4.5 [15]	8 [21]	5 [26]

The average number of publications and the average number of years into the current decade are as follows: Decade 1 (9.7 publications, 5.7 years), Decade 2 (5.5 publications, 3.7 years), and Decade 3 (5.7 publications, 4.2 years). If the surgeons maintain their current publication rates, the expected increases in the number of publications by the end of the decade are factors of 1.76, 2.68, and 2.36, respectively.

Among surgeons currently in their first decade of practice, 8.1% published during premedical education, 90.5% during medical training, and 87.8% in their first decade of practice. In the second decade cohort, 1.4% published during premedical studies, 54.8% during training, 79.5% in their first decade, and 49.3% in their second. Surgeons in their third decade of practice published only during their first (59.1%), second (77.3%), and third (61.4%) decades. Among those in their fourth decade, publication rates increased steadily over time: 2.3% published in their first decade, 52.4% in the second, 69.0% in the third, and 64.3% in the fourth. In contrast, surgeons in their fifth decade of practice demonstrated delayed academic involvement, with no publication activity reported prior to their third decade, during which 53.8% published; this rose to 61.5% in the fourth decade and declined to 38.5% in the fifth.

### Overall Publication Rates and Citation Counts

**Figure 2.**
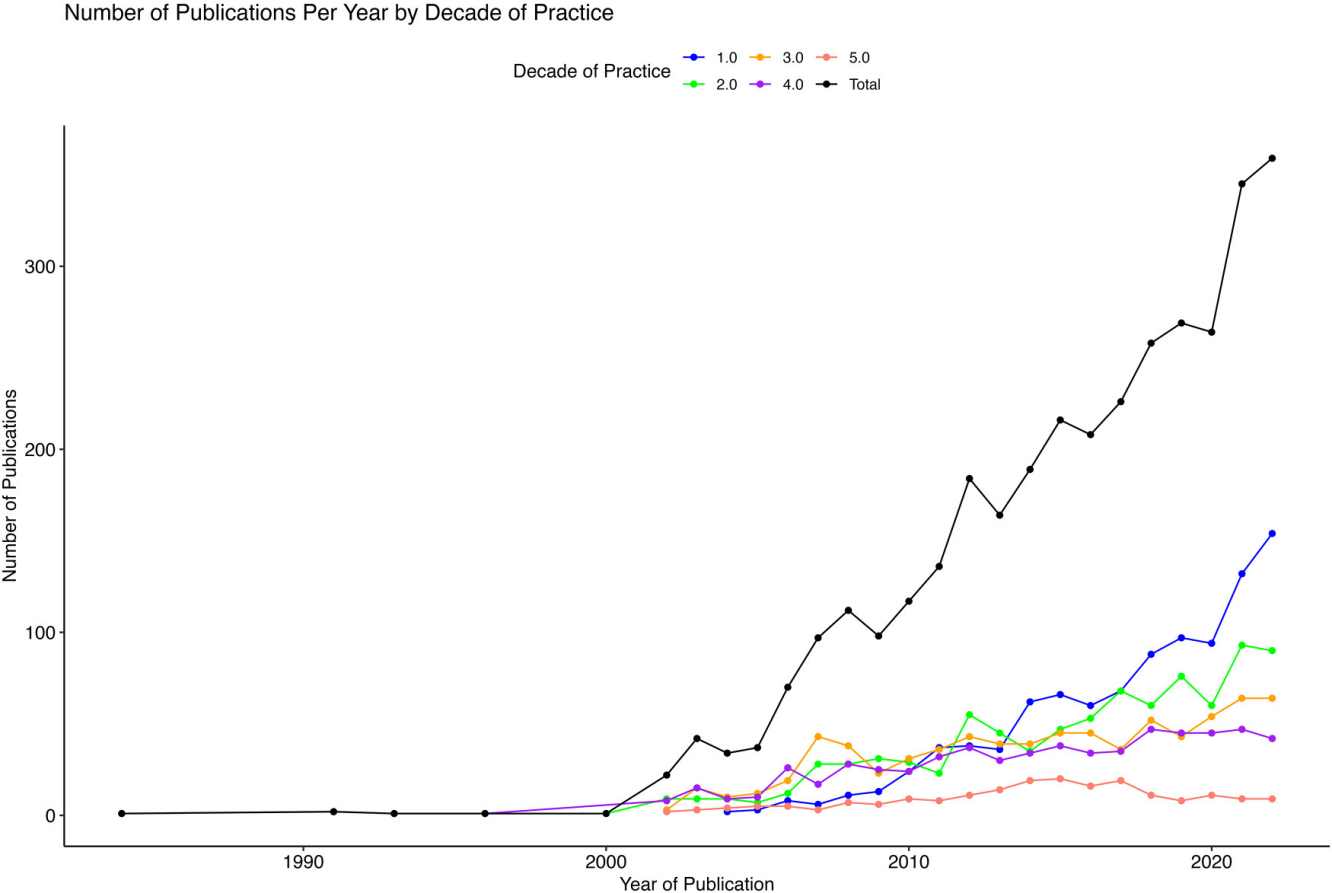
Comparative trends of publications over time by current decade of practice and overall.

Figure 2 demonstrates the trend in the number of unique articles published in a given year. There has been a significant rise in the overall number of publications per year, starting in the year 2000. The mean number of citations per publication, adjusted for the years since publication, for studies published in a given year has remained stable (Suppl. Figure 1). Surgeons have an average of 22 citations per paper (95% CI 0, 64.1).

### Authorship Position Throughout Career

**Figure 3.**
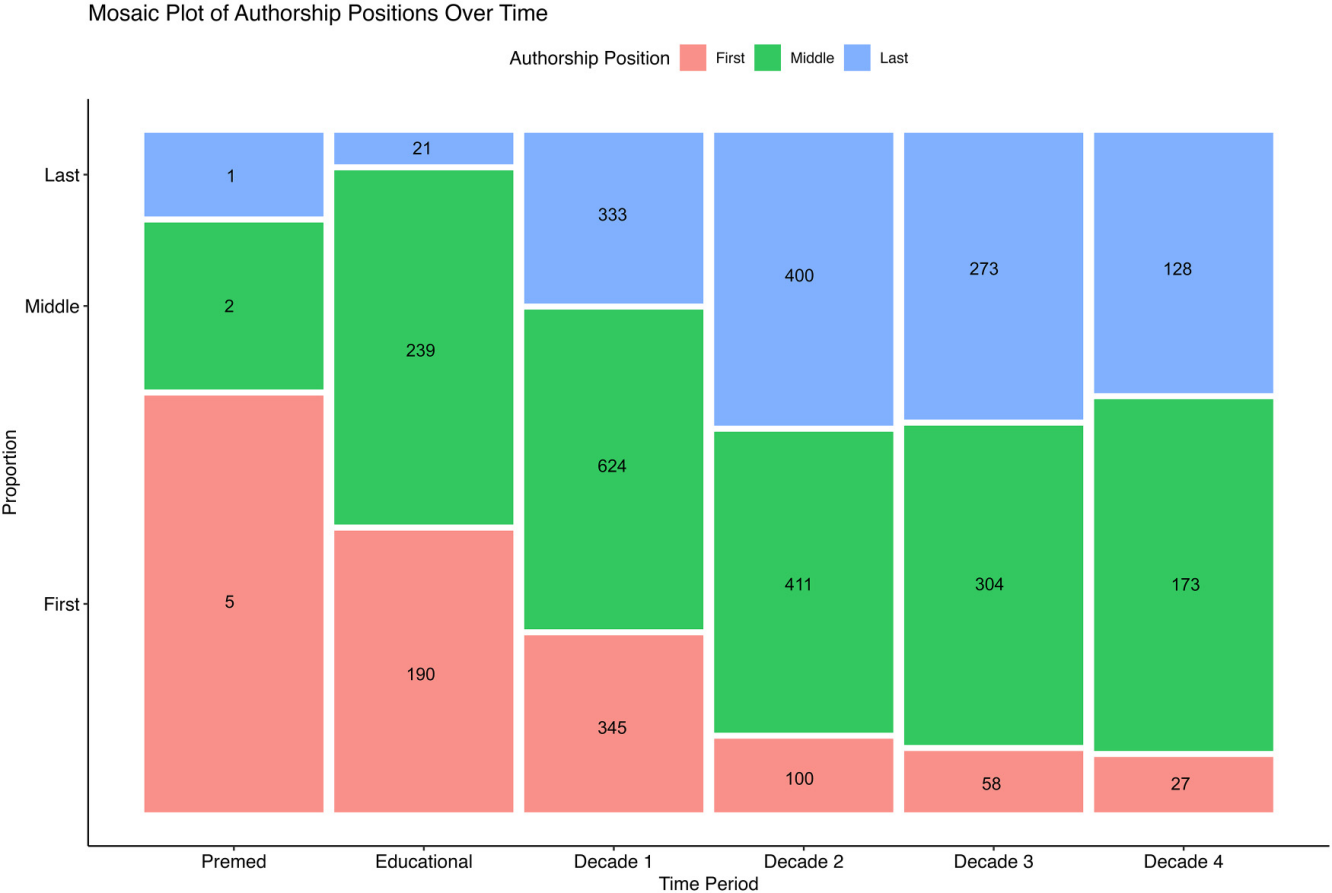
Proportion (N = Count) of first, middle, and last authorship as a function of decade of career. The last author article in the “premed” category represents a two-author study with the staff physician as the first author.

There was a significant change in authorship position found throughout the surgeons’ careers (p < .001, Figure 3) from a larger proportion of first authorship early in the surgeon's careers, to a larger proportion of middle and last authorships later in the surgeon's careers. Post-hoc analysis showed significant differences (p < .001) in proportions between all decades (Figure 3) except the following pairs: The education and pre-medicine decade (p = 1), pre-medicine and decade 1 (p = 1), decade 2 and decade 3 (p = .1), decade 2 and decade 4 (p = .71), as well as decade 3 and decade 4 (p = 1).

### Journals

Most articles were published in Plastic and Reconstructive Surgery (n = 442), Plastic Surgery (n = 265), Plastic and Reconstructive Surgery - Global Open (n = 125), Journal of Plastic Reconstructive and Aesthetic Surgery (n = 103), and Annals of Plastic Surgery (n = 90). Table 2 outlines the volumes of the top 20 journals in which articles were published.

**Table 2.** Counts of the Top 20 Journals in Which Unique Studies Were Published.

Journal	Count
PLASTIC AND RECONSTRUCTIVE SURGERY	442
PLASTIC SURGERY	265
PLASTIC AND RECONSTRUCTIVE SURGERY-GLOBAL OPEN	125
JOURNAL OF PLASTIC RECONSTRUCTIVE AND AESTHETIC SURGERY	103
ANNALS OF PLASTIC SURGERY	90
JOURNAL OF CRANIOFACIAL SURGERY	76
JOURNAL OF BURN CARE & RESEARCH	72
HAND (NEW YORK, N.Y.)	67
THE JOURNAL OF HAND SURGERY	63
AESTHETIC SURGERY JOURNAL	56
BURNS	42
CLINICS IN PLASTIC SURGERY	41
JOURNAL OF RECONSTRUCTIVE MICROSURGERY	34
ANNALS OF SURGICAL ONCOLOGY	34
CLEFT PALATE-CRANIOFACIAL JOURNAL	27
HAND CLINICS	25
JOURNAL OF OTOLARYNGOLOGY-HEAD & NECK SURGERY	25
JOURNAL OF SURGICAL EDUCATION	22
MICROSURGERY	22
THE JOURNAL OF CRANIOFACIAL SURGERY	19

## Discussion

Our data brings to light the publication trajectory of a Canadian plastic surgeon over different decades of their career. Our results demonstrate increased and earlier publishing in newer generations, collaboration between Canadian academic plastic surgeons, increased overall volume of publication over time, and a divergence of research productivity among surgeons.

### Discussion of Trajectory Trends

In the premedical and educational phases, publication rates are relatively modest. This trend is anticipated, as surgeons are in the nascent stages of their careers, primarily focusing on training and education. However, a notable inflection point occurs as surgeons advance into their first decade of practice, where we observe an increase in publication rates. In a study by Spake et al (2021) exploring publication trajectories of plastic surgeons in the United States, an inflection point where productivity peaks is seen in the decade following board certification.^2^ This rise is likely attributable to the culmination of training, the initiation of independent practice, and more opportunities for engagement with academic research. Another notable finding of our study is that surgeons in their fourth or fifth decade of practice maintain a consistent median number of publications per decade, even in the current, still incomplete decade. This is in contrast to the traditional canonical productivity curve of academia, which describes a decline in productivity with time.^14^

Surgeons currently in their second and third decades exhibit a pronounced escalation in publication frequency in their first and second decades of practice, respectively. The data suggest that surgeons in these decades are not only contributing substantially to the literature but are also likely collaborating as peak publications occur simultaneously across time between surgeons in different decades of practice.

When interpreting the publication rates of surgeons for a given decade that they are currently in, it is critical to acknowledge that the decade is not finished. Based on the expected publication calculations, these decades of practice are projected to be the most productive of all of their careers.

### Increased Publishing in Newer Generations

There is a trend of increased and earlier academic output in the newer generations of surgeons. This is analogous to what was seen by Spake et al (2021), where younger surgeons had publication arcs earlier in their career and the increase in their total number of publications per year was steeper.^2^ The average number of publications in the pre-medicine and educational period of surgeons who are currently in their first decade of practice exceeds that of surgeons currently in later decades. Moreover, both the average and total output of plastic surgeons who were currently in their first year of practice exceeds that of their seniors at any point in their careers as seen in Figures 1 and 2. It is likely that the trend seen in our findings will be more significant with the newer generation of trainees, as a study by ElHawary et al 2022 found that from 2015 to 2018, plastic surgery applicants to the University of British Columbia had on average 3.2 peer-reviewed publications.^15^

### Mentorship and Collaboration

Our data underscores the collaborative nature of research in Canadian plastic surgery, with evidence of significant co-authorship: 22% of studies had two or more Canadian plastic surgeons as coauthors. Previous research in plastic surgery and other surgical subspecialties has shown that mentorship in research can enhance the research productivity of both mentees and their mentors.^16-18^ When segmenting our data by the surgeons’ current decade of practice, the average number of yearly publications increases at distinct career phases, though these phases temporally overlap. For instance, surgeons currently in their fourth decade observed a surge in their publication rates during their first decade of practice, whereas those currently in their third decade began to publish more prolifically in their educational years. A potential explanation could be that those currently in their fourth decade collaborated with or served as mentors to the cohort currently in their third decade. Figure 2 supports the idea that more experienced surgeons mentor less experienced surgeons, showing that publication rates increased simultaneously across time for surgeons at all career stages.

The observed shift from first authorship to last authorship among plastic surgeons over successive decades indicates a natural progression from early career hands-on research contributions to a more supervisory and mentorship role in later years. The identified evolution in authorship roles underscores the ongoing commitment to scholarly activity and knowledge dissemination, as seasoned surgeons guide and shape research projects, reflecting a maturing academic career and the nurturing of new talent in the field. To further support this progression, institutions should implement programs that sustain research productivity and foster collaboration, such as formal mentorship initiatives.^16-18^

### Volume and Quantity of Publishing Over Time

The present study demonstrates a large increase in the number of publications per year since 2000 as seen in Figure 2. The increase in research output may be attributed to several factors, including a greater impact of research on career advancement, a trend toward subspecialization, greater research funding, heightened collaboration, improved technologies, and digitization of the publishing process.^5,6,12,13,19-21^ This trend is not confined to plastic surgery. A comprehensive study of the SCOPUS database revealed a significant increase in publication growth following 2004 on top of the already exponential rise observed since 1900.^22^ Of note, there is significant discussion in the literature around the idea that while the number of publications has increased, the quality and impact have decreased.^23-23^ Despite the increased publishing seen in our data, the mean number of citations per publication, adjusted for the years since publication, remained stable.

### Publication Activity Over Time

Our findings demonstrate a growing variation in publication activity, with a broader dispersion of publication rates. Across the data, the confidence intervals—indicative of variability within each decade—widen as the surgeons progress in their careers as seen in Figure 1. This finding signals a divergence in research productivity, with some surgeons likely sustaining or intensifying their research endeavours while others may have decreased their academic engagement. This is illustrated by Suppl. Figure 2, which shows a left-skewed histogram with a peak of 0 publications by senior plastic surgeons, while other senior surgeons have over 40 publications within their third decade of practice. One factor that may contribute to this finding is the attrition from academic to cosmetic practice over time.^26^

A significant portion of Surgeons were found not to participate in research, with at least 36% not publishing in a given decade. Surgeons who are not actively engaged in research represent a valuable knowledge base that could be leveraged through mentoring and collaborating with medical students, residents, and junior faculty. The observed divergence in publication trends reinforces the necessity for institutions to implement policies that not only promote an academic culture but also carefully consider the academic inclinations of surgeons. Moreover, the divergence makes a case for the requirement of positive incentives to be in place for surgeons to continue academic activities later in their careers.

### Limitations and Future Directions

In this study, we only included academic plastic surgeons and findings cannot be applied to surgeons in a non-academic setting. There is a possibility of inadvertently including studies by plastic surgeons who share names with other authors or excluding some studies due to missed prior institutional affiliations. Our search was limited to just two databases, which means we potentially missed studies that were not indexed in these databases. However, this limitation is acceptable for our purposes as our discussion focuses on trends rather than specific numbers. In addition, this study did not account for research years, gaps in training, or advanced academic degrees obtained during the analyzed years, which may have influenced our findings. Our definition of the educational decade may also have underestimated the contribution of residency and fellowship periods to research productivity, as some works may have only been published more than a year after residency or fellowship. Another limitation is that our assessment of publication impact and quality was limited to citation count. Future studies should investigate the quality and disruptiveness of Plastic Surgery publications and the publication trajectories of current medical trainees once they become practicing surgeons. Other areas of future research could investigate publication rates and authorship position by gender.

## Conclusions

The study's findings delineate the trajectory of publication rates among plastic surgeons, revealing an initial modest output during the nascent stages of training that surges as surgeons transition into independent practice. Publication rates continue to increase from decade to decade, with a trend for earlier and increased publications among newer generations of surgeons. Collaborative efforts in research are pronounced, as evidenced by significant co-authorship, with mentorship likely playing a crucial role in shaping the publication trajectories of different career stages. A divergence in research output can be seen in more senior plastic surgeons. Canadian plastic surgeons are publishing significantly in the Canadian journal, *Plastic Surgery,* shaping the national discussion in the field. Incentives must be put in place so that as surgeons gain seniority, they continue to participate and mentor in research.

## Supplemental Material

sj-docx-1-psg-10.1177_22925503251371051 - Supplemental material for Publication Trajectories of Today's Canadian Academic Plastic Surgeons: A Bibliometric Analysis Supplemental material, sj-docx-1-psg-10.1177_22925503251371051 for Publication Trajectories of Today's Canadian Academic Plastic Surgeons: A Bibliometric Analysis by Norbert Banyi, Theresa Buchel, Young Ji Tuen, Sophia Shayan, Rebecca Courtemanche and Jugpal S. Arneja in Plastic Surgery
