# Supplementary material for: Publication Trajectories of Today's Canadian Academic Plastic Surgeons: A Bibliometric Analysis
Source: Plast Surg (Oakv). 2025 Aug 29:22925503251371051. Online ahead of print. doi: 10.1177/22925503251371051 (PMC12397090; doi:10.1177/22925503251371051)
Supplement: sj-docx-1-psg-10.1177_22925503251371051 - Supplemental material for Publication Trajectories of Today's Canadian Academic Plastic Surgeons: A Bibliometric Analysis [file sj-docx-1-psg-10.1177_22925503251371051.docx]

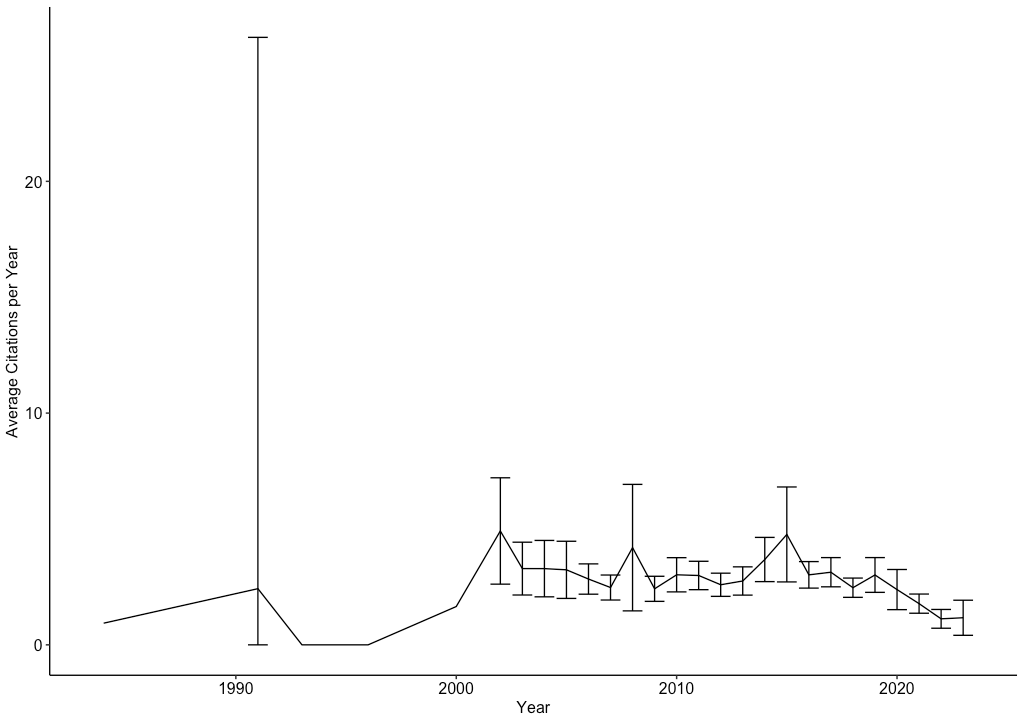


**Suppl. Figure 1:** Mean number of citations per publication, adjusted for the years since publication, for studies published in a given year. Vertical bars represent 95% confidence intervals.


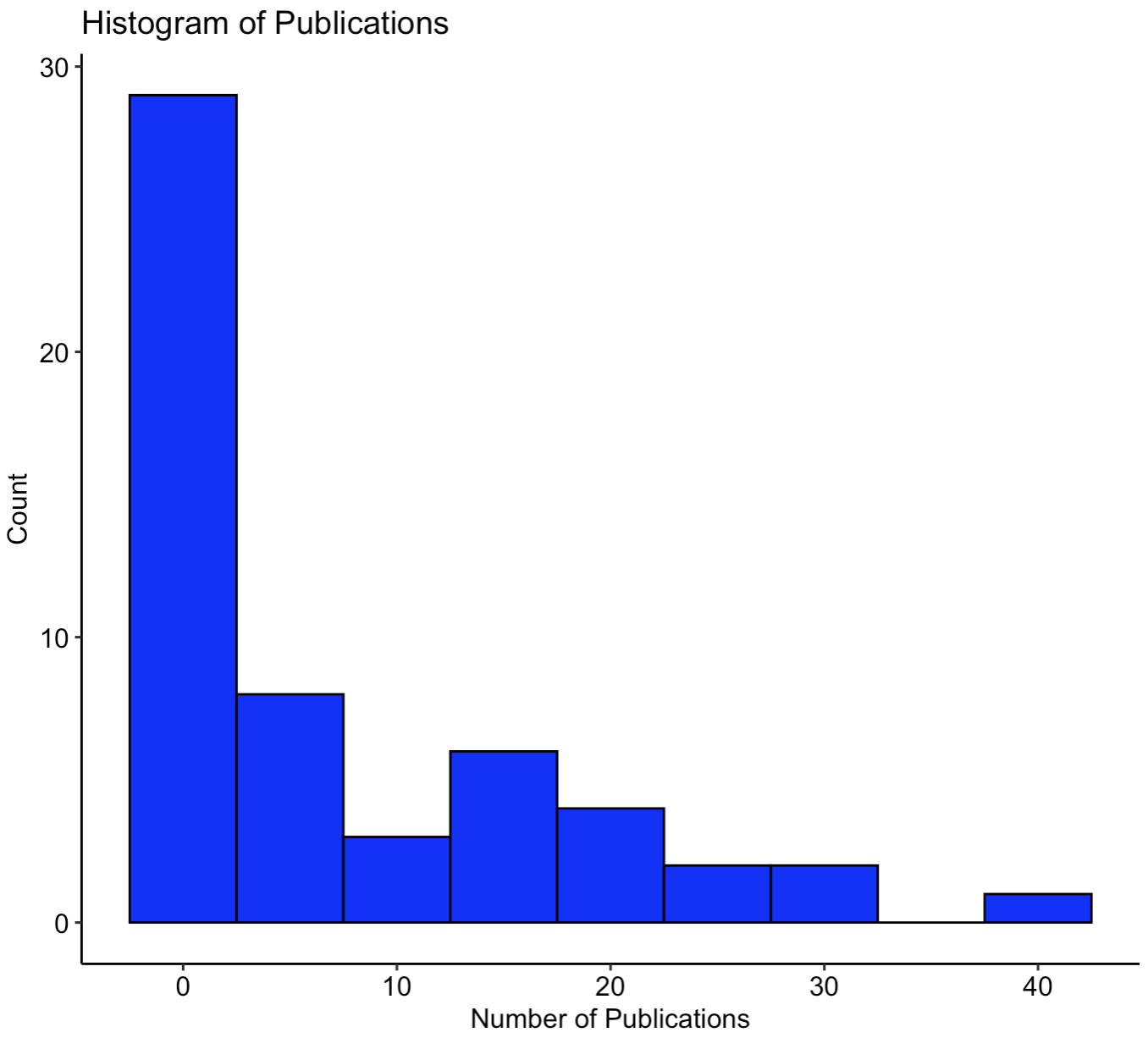


**Suppl. Figure 2:** Histogram of the number of plastic surgeons currently beyond their third decade of practice versus their number of publications during the third decade.

**Suppl. Table 1:** Results (p-values) of post-hoc Dunn’s tests between surgeons grouped based on the current decade for periods that reached significance on Kruskal-Wallis testing.

| **Pre-Med Decade (H: 10.8, p = 0.01)** | | | |
| --- | --- | --- | --- |
| **Decade of Practice (median, IQR)** | Current Decade 1 (0, 0) | Current Decade 2 (0,0) | Current Decade 3 (0,0) |
| Current Decade 2 (0,0) | **0.045*** |  |  |
| Current Decade 3 (0,0) | **0.032*** | 1 |  |
| Current Decade >4 (0,0) | **0.019*** | 1 | 1 |
| **Educational Decade (H 147.1, p <0.001)** | | | |
| **Decade of Practice (median, IQR)** | Current Decade 1 (3, 5) | Current Decade 2 (1, 2) | Current Decade 3 (0,0) |
| Current Decade 2 (1,2) | **<0.0001*** |  |  |
| Current Decade 3 (0,0) | **<0.0001*** | **<0.0001*** |  |
| Current Decade >4 (0,0) | **<0.0001*** | **<0.0001*** | 1 |
| **Decade 1 (H 100.6, p < 0.001)** | | | |
| **Decade of Practice (median, IQR)** | Current Decade 1 (6.5, 11.75) | Current Decade 2 (3, 6.25) | Current Decade 3 (1, 3.25) |
| Current Decade 2 (3, 6.25) | **0.0364*** |  |  |
| Current Decade 3 (1, 3.25) | **<0.0001*** | 0.0629 |  |
| Current Decade >4 (0,0) | **<0.0001*** | **<0.0001*** | **0.0001*** |
| **Decade 2 (H 15.6, p <0.001)** | | | |
| **Decade of Practice (median, IQR)** | Current Decade 2 (0.5, 6) | Current Decade 3 (3, 11.25) |  |
| Current Decade 3 (3, 11.25) | **0.008*** |  |  |
| Current Decade >4 (0, 3) | 0.221 | **0.0001*** |  |
| **Decade 3 (H 0.44, p =0.51)** | | | |
